# Microalgal Metabolic Network Model Refinement through High-Throughput Functional Metabolic Profiling

**DOI:** 10.3389/fbioe.2014.00068

**Published:** 2014-12-10

**Authors:** Amphun Chaiboonchoe, Bushra Saeed Dohai, Hong Cai, David R. Nelson, Kenan Jijakli, Kourosh Salehi-Ashtiani

**Affiliations:** ^1^Division of Science and Math, New York University Abu Dhabi, Abu Dhabi, UAE; ^2^Center for Genomics and Systems Biology (CGSB), New York University Abu Dhabi Institute, Abu Dhabi, UAE; ^3^Engineering Division, Biofinery, Manhattan, KS, USA

**Keywords:** microalgae, *Chlamydomonas reinhardtii*, flux balance analysis, phenotype microarray, metabolic network refinement

## Abstract

Metabolic modeling provides the means to define metabolic processes at a systems level; however, genome-scale metabolic models often remain incomplete in their description of metabolic networks and may include reactions that are experimentally unverified. This shortcoming is exacerbated in reconstructed models of newly isolated algal species, as there may be little to no biochemical evidence available for the metabolism of such isolates. The phenotype microarray (PM) technology (Biolog, Hayward, CA, USA) provides an efficient, high-throughput method to functionally define cellular metabolic activities in response to a large array of entry metabolites. The platform can experimentally verify many of the unverified reactions in a network model as well as identify missing or new reactions in the reconstructed metabolic model. The PM technology has been used for metabolic phenotyping of non-photosynthetic bacteria and fungi, but it has not been reported for the phenotyping of microalgae. Here, we introduce the use of PM assays in a systematic way to the study of microalgae, applying it specifically to the green microalgal model species *Chlamydomonas reinhardtii*. The results obtained in this study validate a number of existing annotated metabolic reactions and identify a number of novel and unexpected metabolites. The obtained information was used to expand and refine the existing COBRA-based *C. reinhardtii* metabolic network model iRC1080. Over 254 reactions were added to the network, and the effects of these additions on flux distribution within the network are described. The novel reactions include the support of metabolism by a number of d-amino acids, l-dipeptides, and l-tripeptides as nitrogen sources, as well as support of cellular respiration by cysteamine-*S*-phosphate as a phosphorus source. The protocol developed here can be used as a foundation to functionally profile other microalgae such as known microalgae mutants and novel isolates.

## Introduction

Optimization of algal metabolism toward improved bioproduct production while maintaining strain robustness remains a challenge that requires experimental strategies informed through systems-level analyses of metabolism. The use of metabolic network models can guide the development of optimization strategies that would be otherwise difficult through rational designs (Oberhardt et al., 2009; Schmidt et al., 2010; Koskimaki et al., 2013; Koussa et al., 2014). While an increasing number of algal species are being isolated and sequenced for biofuel or other applications, to date, there are only a handful of reconstructed algal networks available (Koussa et al., 2014). A major obstacle in the reconstruction of high-quality network models for algae remains hinged on the inability to obtain rapid and high-throughput metabolic phenotypic data to guide and validate reconstruction efforts.

One potential high-throughput phenotypic analysis technology is the Biolog OmniLog^®^ phenotype microarray (PM) (Biolog, Hayward, CA, USA) (Bochner et al., 2001; Bochner, 2003, 2009). By assaying cellular metabolism in response to thousands of metabolites, signaling molecules, and effector molecules (as well as osmolites), the Biolog PM assays have greatly boosted functional metabolic profiling by providing insight into function, metabolism, and environmental sensitivity (Bochner et al., 2001; Bochner, 2003, 2009). Biolog PM assays rely on the measurement of metabolite utilization of cells in 96-well microplates. Each well contains different nutrients, metabolites, and pH and osmolarity solutes. Other bioactive molecules such as antibiotics and hormones may also be assayed. This utilization is assessed and measured in the form of cell respiration determined by the amount of color development produced by the NADH reduction of a tetrazolium-based redox dye (Bochner et al., 2001; Bochner, 2003, 2009). Plates can be monitored automatically over time with the OmniLog platform. A common set of 20 96-well microplates are designed to measure carbon, nitrogen, sulfur, phosphorus utilization phenotypes, along with osmotic/ion, and pH effects. This high-throughput and standardized approach has the ability to provide a quick method for the phenotypic comparison of different strains and organisms in a convenient manner leading to insights into the metabolic state of the cell. While the PM technology has been used for metabolic phenotyping of various microbial species including bacteria and fungi, it has not been reported for the phenotyping of microalgae. Likewise, the technology has been successfully used for verification and expansion of a number of existing microbial metabolic network models (Bochner et al., 2001; Bochner, 2003, 2009; Bartell et al., 2014), yet its use for improvement of microalgal models remains unreported.

The goal of the present study is to establish a reliable method for characterizing metabolic phenotypes of microalgae that can be used to expand existing network models or guide the reconstruction of new algal metabolic models. We present the implementation of the PM platform for metabolic phenotyping of microalgae using *Chlamydomonas reinhardtii* as a model organism then expand a well-curated existing metabolic network model of *C. reinhardtii* accordingly.

## Materials and Methods

### Phenotype microarray experiments

Phenotyping was done using standard Biolog assay plates and using the OmniLog instrument. In total 190 substrate utilization assays for carbon sources (PM01 and PM02), 95 substrate utilization assays for nitrogen sources (PM03), 59 nutrient utilization assays for phosphorus sources, and 35 nutrient utilization assays for sulfur sources (PM04), along with peptide nitrogen sources (PM06–08) were utilized. A defined tris-acetate-phosphate (TAP) medium (Gorman and Levine, 1965) containing 0.1% tetrazolium violet dye “D” (Biolog, Hayward, CA, USA) was used for the PM tests. The carbon, nitrogen, phosphorus, or sulfur component of the media was omitted from the defined medium when applied to the respective PM microplates that tested for each of those sources.

*Chlamydomonas reinhardtii* strain CC-503 was obtained from the *Chlamydomonas* Resource Center at the University of Minnesota, USA. Cells were grown in fresh TAP media to mid-log phase, then spun down at 2,000 × *g* for 10 min, and then resuspended in fresh media to a final concentration of 1 × 10^6^ cells before inoculation into Biolog’s 96-well plates. A 100 μL aliquot of cell-containing media was inoculated into each well before the plates were inserted into the OmniLog system. A final concentration of 400 μL/mL timentin^®^ (GlaxoSmithKline, New Zealand) was used to inhibit bacterial growth in all plates. In addition, ampicillin and kanamycin were used at 50–100 μg/ml occasionally. Bacterial contamination was monitored by streaking cells on yeast extract/peptone plates and performing gram stains before and after Biolog assays. All microplates were incubated at 30°C for up to 7 days and the dye color change (in the form of absorbance) was read with the OmniLog system every 15 min. As the OmniLog instrument does not provide a source of continuous light during incubation, the algae is assumed to be carrying out heterotrophic respiration.

### Data analysis

The Biolog PM data analysis was carried out using an OmniLog phenotype microarray (OPM) software package (Vaas et al., 2012, 2013) that runs within the R software environment. The raw kinetic data were exported as CSV files to the OPM package and then the biological information was added as metadata (e.g., strain designation, growth media, temperature, etc.). Kinetic curves were plotted from the raw data in the form of *xy* and level plots, and a statistical analysis was carried out to visualize the metabolic properties and generate OmniLog values. An OmniLog value or the curve parameter “A” simply lists the maximum height of the growth curve.

Duplicate assays were carried out for all the plates that were tested to assess reproducibility of the data. An assay was considered positive when the absorbance (OmniLog value) was positive after subtraction from the negative control well and the respective blank well. This summation is a representation of the abiotic reaction of the dye with the media in the presence of the tested compound.

### Identification of reactions and genes associated with new metabolites

Gene to reaction associations for compounds were established as follows: assignment of a compound’s enzyme commission number (EC) and relevant reactions were performed by searching KEGG^1^ and MetaCyc^2^. The genomic evidence for each reaction was then recovered by using the identified EC numbers as a search basis in multiple available annotation resources from available algal annotation databases, such as the Joint Genome Institute (JGI), Phytozome^3^, and peer-reviewed publications. When the query returned no genomic evidence for a given EC number, the relevant associated proteins in other organisms were identified then a profile-based search was carried out using the NCBI PSI-BLAST server with default settings and using non-redundant protein sequences (nr) in *C. reinhardtii* (taxid: 3055). PSI-BLAST hits with E values of ≤0.05 were manually curated for relevance to the searched EC number through either the evaluation of their described enzymatic activity, or by querying those BLAST hits through EMBL-EBI Pfam^4^, or InterPro^5^ protein domain prediction servers.

### Model refinement and evaluation

Identified reactions with their associated genes were added to iRC1080 using the COBRA Toolbox functions add Reaction and Change Gene Association. In addition, transport reactions for the new metabolites were incorporated into the model as transport by passive diffusion from the extracellular medium into the cytosol. The behavior of the new resultant model, iBD1106, was tested by carrying out flux balance analyses under light and dark conditions for the maximization of biomass as the objective function. The comparison of the two models was based on reported shadow prices (sensitivity of the objective function to changes in system variables) of the metabolites. The Biomass function was defined previously (Chang et al., 2011) for growth under dark and light conditions. The revised model can be found in the supplementary file iBD1106.xml in an SBML file format.

## Results

### Phenotype microarray screening of model alga *Chlamydomonas reinhardtii*

To implement the use of the PM platform for algal metabolic phenotyping, we used *C. reinhardtii* as a model. The single-cell green alga *C. reinhardtii* is a model organism that has been widely used for basic and applied biological research. Its genome was sequenced and publically released by JGI in 2007 (Merchant et al., 2007) and genome-scale models of its metabolism have been reconstructed (May et al., 2009; Chang et al., 2011; Dal’Molin et al., 2011). The ability to grow phototrophically or heterotrophically, along with rapid growth and scalability, are features that make this alga an attractive model system for algal-based biofuel studies.

**Figure 1:**
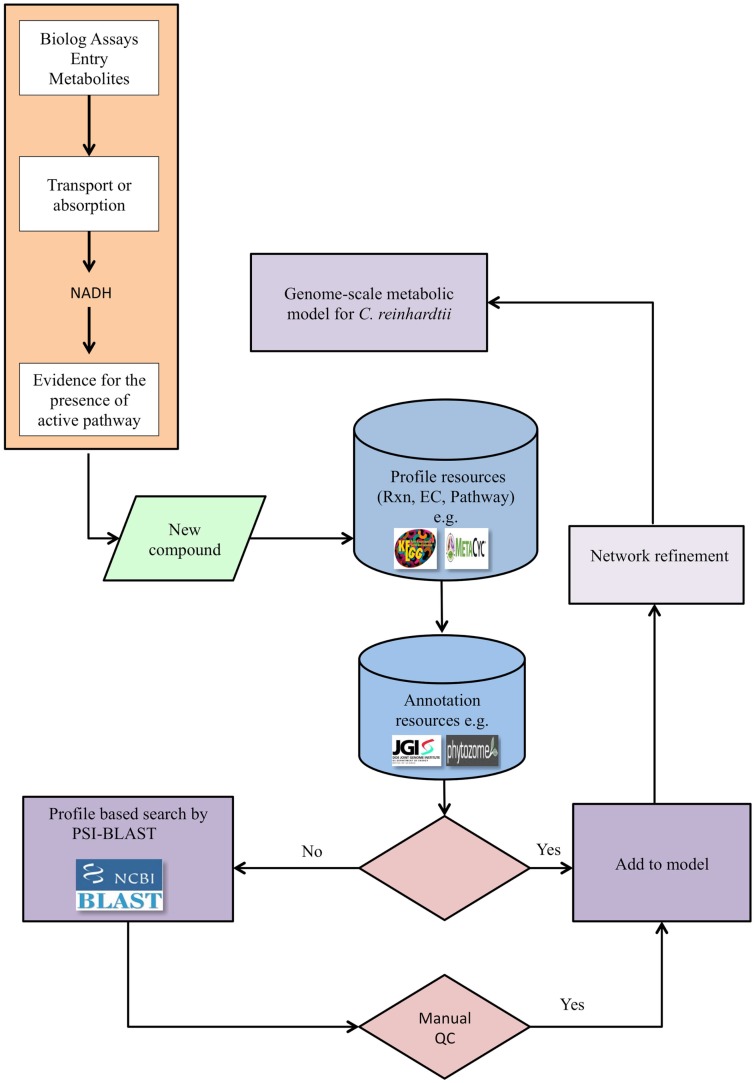
**The pipeline for genome-scale metabolic network refinement using PM data**. After a new compound tests positive in a PM assay, its enzyme commission number (EC), reaction, and pathway are identified from available databases, e.g., KEGG and MetaCyc. Genomic evidence is then extracted directly from genomic and annotation resources when available and constitutes a link between genotype and phenotype. When direct genomic evidence is unavailable, the protein sequence is identified from the EC numbers and through the protein sequence, genetic evidence is identified via PSI-BLAST. The reconstructed metabolic network is then refined based on newly identified compounds, but only after a quality control step. The quality control step entails querying the protein domains using relevant databases.

Our pipeline (Figure 1) integrates the high-throughput PM assays, applied to the alga of interest, with genomic searches to provide experimental evidence that can lead to the refinement of an existing metabolic network model. The pipeline may also be applied for a new reconstruction if an existing model is not available. The PM assays test the ability of the alga to utilize various carbon, nitrogen, sulfur, and phosphorus sources in a minimal medium. When a new compound tests positive for utilization, the compound’s relevant reaction profiles are defined using metabolic knowledge bases such as KEGG (see text footnote 1) or MetaCyc (see text footnote 2). This step defines all potential reactions and pathways that can be associated with a metabolite to provide EC numbers. The next step is to find supporting genetic evidence from genetic databases specific to the alga, such as databases from the JGI, Phytozome (see text footnote 3), or peer-reviewed publications. If genetic evidence is available, the reactions and metabolites are added to the model to expand and refine the model. If, on the other hand, genomic evidence is not found in support of the EC number, a profile-based search, such as PSI-BLAST, can be performed to identify candidate genes associated with the reaction. The results of such searches are then manually evaluated; those passing this QC step are added to the network model. In exceptional cases, if genes are not identified for reactions but compelling biochemical evidence exists, reactions may be provisionally added to the network pending future investigations.

### Implementation and validation

**Figure 2:**
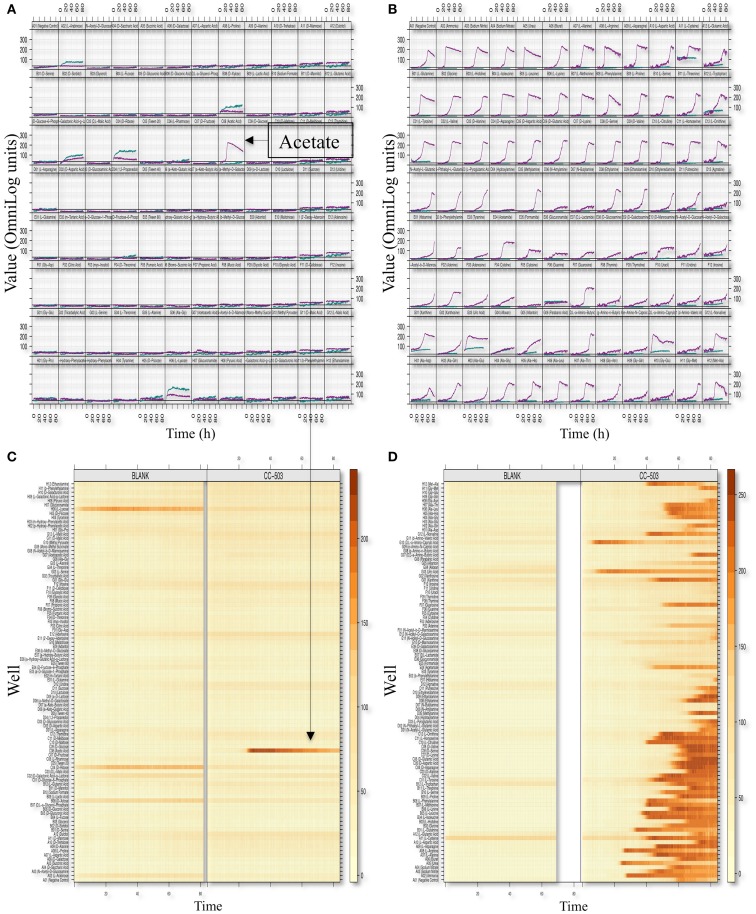
**Phenotypic microarray profiling selection of *C. reinhardtii***. Respiration (or growth) *xy*-plots and level plots of the PM01 [Carbon sources; **(A,B)**] and PM03 [Nitrogen sources; **(C,D)**] assay plates are shown. The figure is an 8 × 12 array where each cell represents a well plate and, thus, a given metabolite or growth environment. Within each cell or well representation, curves represent dye conversion by reduction (*y*-axis) as a function of time (*x*-axis). PM respiration curves from the CC-503 and blank are both shown in each cell and are indicated by color (teal color represents blank and purple color represents CC-503). The level-plot represents each respiration curve as a thin horizontal line changing color (or remaining unchanged) over time. Shading color changes from light yellow to dark orange or brownish based on the level of respiration measurement values, with the brownish color representing higher respiration values. Metabolites utilized by *C. reinhardtii* (CC-503) and the blank plates are shown.

We optimized the PM assays for metabolic profiling of *C. reinhardtii* by modifying the standard Biolog protocol with respect to inoculum concentration, type of dye, and pre-inoculation growth conditions (Materials and Methods). We used plates 1–4 and 6–8 of the PM platform, which provide a range of test compounds including utilization of carbon, nitrogen, sulfur, phosphorus, and a variety of di- and tripeptides. The summary kinetics of selected plates (PM01 and PM03) are shown in Figure 2. Splined-based curve fitting was implemented to extract the curve parameters [the lag phase (λ), the respiration (or growth rate μ or the steepness of the slope), the maximum cell respiration “A,” and the area under the curve (AUC)]. The maximum cell respiration “A” of the blank and negative controls of each microwell plate (which represents abiotic reactivity of the dye with the medium and the test metabolite) were used as background subtraction values to identify positive metabolites. The “*xy*-plots” show the respiration measurements over time mapped to the assay 96-well plates, in terms of the raw measurements values (*y*-axis) and time (*x*-axis). In addition, the data was transformed to a heat map format to allow for a quick comparative overview of the multitude of the kinetic data. The heat map presents the kinetic values with different colors (varied from light yellow to dark orange or brownish; Figures 2B,D).

**Figure 3:**
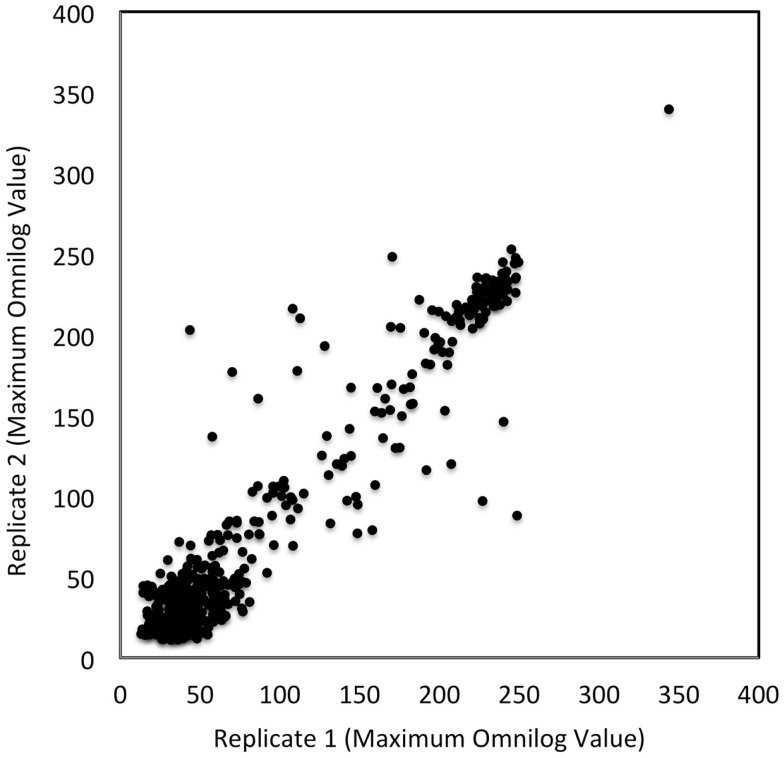
**Reproducibility of PM tests**. OmniLog values were collected over a 168 h period and the maximum values were plotted for two replicate studies. Each axis represents the maximum OmniLog values for each study (the *x*-axis being one replicate study and the *y*-axis another). Identical values fall on a 45° line; there are a few deviating test values (some deviations were by more than 50 units). Each point represents a single maximum OmniLog value.

To assess the level of combined experimental and biological noise and systematic errors and biases from Biolog’s PM measurements, the data from two independent replicate experiments were plotted against one another (Figure 3). This figure visually assesses the reproducibility of the PM data obtained from PM01–04 and PM10 plates. Figure 3 shows that the majority of the data were identical as they fall on the 45° line with only a few outliers. This plot confirms the quality and reproducibility of the experiments for this alga.

### Identification of new metabolites

**Figure 4:**
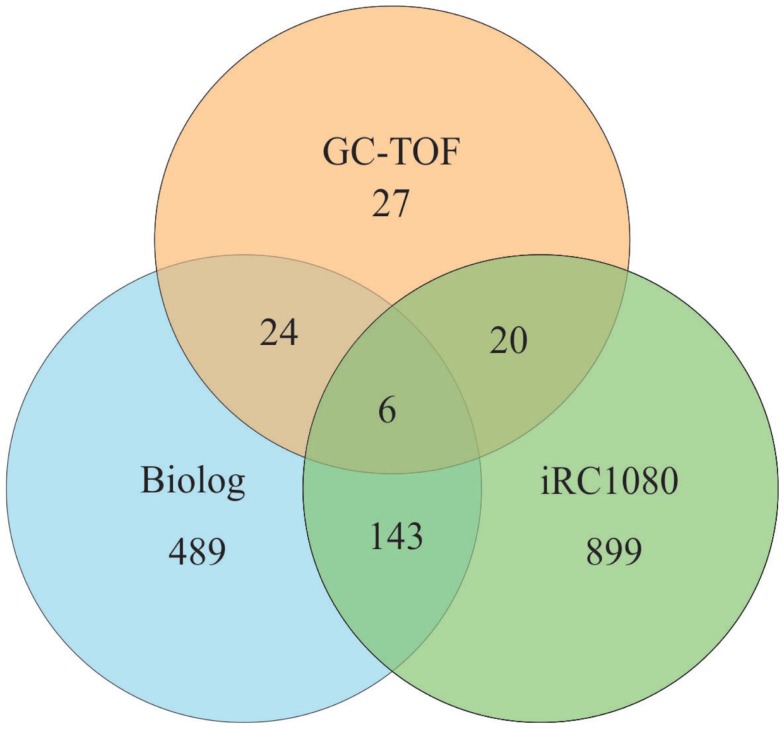
**Venn diagram of metabolites**. The Venn diagram is a representation of metabolites common to Biolog’s PM plates, the iRC1080 metabolic model and Gas Chromatography time-of-flight (GC-TOF) experiments. Each circle indicates the total number of metabolites that exists in each respective method of study, while the overlapping regions represent the number of metabolites shared between those methods of study. The iRC1080 metabolic model contains a total of 1,068 unique metabolites, the GC-TOF identified a total of 77 metabolites (Bölling and Fiehn, 2005), while there are a total of 662 metabolites tested using Biolog’s PM plates.

We compared the number of metabolites that can be identified by Biolog’s PM (662 chemical compounds from seven plates {PM01–PM04, and PM06–PM08}) with the iRC1080 metabolites and the metabolites measured using gas chromatography time-of-flight (GC-TOF) (Bölling and Fiehn, 2005) (Figure 4). Only six metabolites were overlapping among the three sets (adenine, glycerol, glycine, myo-Inositol, putrescine, and uracil), while 149 were common between iRC1080 and the Biolog set under investigation. This shows that while each technology/tool has its strength in metabolic profiling research, the Biolog set can be a significant source of new metabolic information.

After subtracting the background signal, we observed acetic acid as the only positive assay for carbon utilization (in PM01 plate). Detection of acetate as the only carbon source from this plate is consistent with the *Chlamydomonas* literature (e.g., Harris, 2009) and provides evidence for specificity of our assays. Four positive reactions for sulfur utilization (sulfate, thiosulfate, tetrathionate, d,l-Lipoamide) and four positive assays for phosphorus utilization (thiophosphate, dithiophosphate, d-3-phospho-glyceric acid and cysteamine-*S*-phosphate) were detected. *C. reinhardtii* showed positive results for several nitrogen sources including both l-amino and d-amino acids, and less common amino acids such as l-homoserine, l-pyroglutamic acid, methylamine, ethylamine, ethanolamine, and d,l-α-amino-butyric acid. Furthermore, a large number of dipeptides and a few tripeptides assayed positive (Table 1).

**Table 1:** **List of identified positive substrate utilization metabolites (C, P, S, N) not present in the iRC1080 model**.

Biolog chemical	EC^a^	Gene annotation	PSI-BLAST
Cysteamine-*S*-phosphate	3.1.3.1	JLM_162926^b^^,^^c^^,^^d^^,^^e^	
Tetrathionate	1.8.2.2		Insignificant E-value
	1.8.5.2		Insignificant E-value
d-Alanine	1.4.1.1		XP_001700222.1
	1.5.1.22		Failed manual QC
	2.1.2.7		Insignificant E-value
	1.4.3.3	Cre02.g096350.t1.3^f^	
	2.3.2.10		Insignificant E-value
	2.3.2.14		Insignificant E-value
	2.3.2.16		Insignificant E-value
	2.3.2.17		Insignificant E-value
	2.3.2.18		Insignificant E-value
	2.6.1.21		Failed manual QC
	3.4.13.22		XP_001698572.1, XP_001693532.1, XP_001701890.1, XP_001700930.1
	3.4.16.4	Chlre2_kg.scaffold_ 14000039^b^^,^^c^^,^^d^	
	3.4.17.8		Failed manual QC
	3.4.17.13		Insignificant E-value
	3.4.17.14		Insignificant E-value
	4.5.1.2		Insignificant E-value
	6.1.1.13		Failed manual QC
	6.1.2.1		Failed manual QC
	6.3.2.4	au.g14655_t1^b^^,^^c^^,^^d^	
	6.3.2.10		Failed manual QC
	6.3.2.16		Insignificant E-value
	6.3.2.35		Insignificant E-value
d-Asparagine	1.4.5.1		Insignificant E-value
	1.4.3.3	Cre02.g096350.t1.3^f^	
	3.1.1.96		Insignificant E-value
	2.3.1.36		Insignificant E-value
	1.4.99.1		XP_001692123.1
	3.5.1.77	e_gwW.1.243.1^b^^,^^c^	
	3.5.1.81		Insignificant E-value
	5.1.1.10		Failed manual QC
d-Aspartic acid	6.3.1.12		Insignificant E-value
	1.4.3.3	Cre02.g096350.t1.3^f^	
d-Glutamic acid	1.4.3.7		Insignificant E-value
	1.4.3.3		Insignificant E-value
d-Lysine	5.4.3.4		Insignificant E-value
	1.4.3.3	Cre02.g096350.t1.3^f^	
	6.3.2.37		Failed manual QC
d-Serine	2.7.11.8		Insignificant E-value
	2.7.11.17	Cre12.g486350. t1.3^b^^,^^c^^,^^d^^,^^e^	
	3.4.21.78		Failed manual QC
	3.4.21.104		Failed manual QC
	4.3.1.18	g6244.t1^e^	Failed manual QC
	6.3.2.35		Insignificant E-value
	6.3.3.5		Insignificant E-value
	1.4.3.3	Cre02.g096350.t1.3^f^	
d-Valine	1.21.3.1		Failed manual QC
	6.3.2.26		Failed manual QC
	1.4.3.3	Cre02.g096350.t1.3^f^	
l-Pyroglutamic acid			
Thiophosphate			
Dithiophosphate			
Ethylamine	6.3.1.6		
d,l-α-Amino-butyric acid	2.1.1.49		Insignificant E-value
	1.4.3.3	Cre02.g096350.t1.3^f^	
Di-peptide	3.4.13.18	Cre02.g078650.t1.3^b^	
Tri-peptide	3.4.11.4	Cre16.g675350.t1.3^b^	

*^a^Reaction was not include if no gene was identified*.

*^b^Phytozome version 10.0.2 (http://phytozome.jgi.doe.gov/pz/portal.html#!info?alias=Org_Creinhardtii)*.

*^c^JGI version 4 (Ghamsari et al., 2011)*.

*^d^Augustus version 5 (Chang et al., 2011)*.

*^e^KEGG (http://www.genome.jp/kegg/kegg1.html)*.

*^f^JGI version 3.1 (Manichaikul et al., 2009)*.

Altogether, we identified 128 new metabolites from the PM data that were not present in our iRC1080 metabolic model: eight d-amino acids, tetrathionate, thiophosphate, dithiophosphate, cysteamine-*S*-phosphate, l-pyroglutamic acid, and ethylamine, 108 dipeptides, and 5 tripeptides. We note that sequence specificity was observed for utilization of both di- and tripeptides. The identified metabolites are summarized in Table 1 and Table S2 in Supplementary Material.

We searched KEGG and MetaCyc to define all possible reactions and EC numbers associated with the identified new metabolites. Forty-nine unique EC numbers were associated with the newly identified metabolites. Table S2 in Supplementary Material includes pathways, reactions, EC numbers, proteins, and *Chlamydomonas* annotation sources for each of the metabolites. Five different sources were used to obtain genomic evidence for the reactions. These included Phytozome Version 10.0.2 (Goodstein et al., 2012), JGI Version 4 (Ghamsari et al., 2011), AUGUSTUS 5.0 and 5.2 (Chang et al., 2011), annotations from Manichaikul et al. (2009), and KEGG (Kanehisa et al., 2014). Out of 49 searched ECs, 15 transcripts could be found with annotations matching the searched ECs (Table 1; Tables S1 and S2 in Supplementary Material).

The metabolic reactions and their respective EC numbers for which no genomic evidence was found (using the aforementioned resources) were then entered into the Universal Protein Resource website (UniProt)^6^ (Apweiler et al., 2004; Consortium, 2014). There, sequences that are related to the metabolites but are from other organisms were identified. Those sequences were then used to run Position-Specific Iterated BLAST (PSI-BLAST queries)^7^ from the NCBI website to identify homologous sequences in *C. reinhardtii*. Only the sequences that produced significant alignments were considered; specifically, results with an E-value below 0.005 were retained. The final step before integrating the genes from the PSI-BLAST results with the iRC1080 metabolic model was to check whether the genes’ relevant reactions related to the new metabolites; only hits with relevant annotated enzymatic reactions were kept. The PSI-BLAST yielded four additional transcripts (Table 1; Table S2 in Supplementary Material).

### Model refinement

The metabolites identified as new to the network were categorized and annotated in the model based on their utilization into nitrogen sources, phosphate sources, and sulfur sources. The nitrogen source metabolites were 8 d-amino acids, 2 l-amino acids, 108 dipeptides, and 5 tripeptides. The phosphate sources were cysteamine-*S*-phosphate, thiophosphate, and dithiophosphate. The only new sulfur source metabolite was tetrathionate. No genomic evidence for tetrathionate was found in databases and its PSI-BLAST E values did not pass the threshold of 0.005, thus, no reaction for this metabolite was added to the model. In addition, l-pyroglutamic acid, thiophosphate, dithiophosphate, and ethylamine were not added to model due to lack of genomic evidence.

To expand the existing model, reactions associated with the new metabolites and the genes associated with the new reactions were added to iRC1080 model to generate an expanded network, iBD1106. iBD1106 accounts for 2,445 reactions, 1,959 metabolites, and 1,106 genes (Table 2). The new 254 added reactions are distributed as follows: 20 amino acid reactions, 108 di-peptide reactions, 5 tri-peptide reactions, and 120 transport reactions (Table 3). The new 20 amino acids reactions were associated with 4 new genes (Cre02.g096350.t1.3, au.g14655_t1, e_gwW.1.243.1, Cre12.g486350.t1.3). The d-amino acids are oxidized into ammonium and a 2-oxo-carboxylate via the following reaction with EC number of 1.4.3.3 and associated gene Cre02.g096350.t1.3:

(1) $$\text{D}-\text{amino acid}+{\text{O}}_{\text{2}}+{\text{H}}_{\text{2}}\text{O}\to \text{N}{\text{H}}_{\text{4}}+{\text{H}}_{\text{2}}{\text{O}}_{2}+\text{2}-\text{oxo carboxylate}$$

**Table 2:** **Contents of iRC1080 and iBD1106**.

Model	Reactions	Metabolites	Genes
iRC1080	2,191	1,706	1,086
iBD1106	2,445	1,959	1,106

**Table 3:** **Summery of new reactions in iBD1106**.

Category or class of reactions	Number of reactions
Amino acids	20
Dipeptides	108
Tripeptides	5
Transport reaction	120

Equation 1 is a general reaction for all d-amino acids. However, some d-amino acids contribute to different reactions in addition to their own oxidation reactions. For example, d-serine reacts with ATP producing ADP and phospho-d-serine. Moreover, the chirality of d-amino acids can also be inverted into L forms and vise versa through annotated racemases (Table S2 in Supplementary Material).

Four genes identified by PSI-BLAST were added into the model and account for the d-alanine transaminase reaction (Eq. 2); XP_001698572.1, XP_001693532.1, XP_001701890.1, XP_001700930.1:

(2) 2−oxoglutarate+D−alanine↔D−glutamate+pyruvate

In addition, XP_001692123.1, a PSI-BLAST identified gene, was associated with the oxidation of d-asparagine reaction as shown in Eq. 1.

A total of 113 added new reactions account for the hydrolysis of dipeptides and tripeptides. The hydrolysis of dipeptides and tripeptides are associated with two genes, one for dipeptides (Cre02.g078650.t1.3), and one for tripeptides (Cre16.g675350.t1.3). The dipeptides and tripeptides are decomposed into their unit l-amino acids, for instance, Leu–Pro decomposes into l-leucine and l-proline.

With respect to sources of phosphorus, a reaction for hydrolysis of cysteamine-*S*-phosphate into cysteamine and phosphate was added according to the following reaction that is associated with the gene JLM_162926:

(3) $$\text{Cysteamine}-\text{S}-\text{Phosphate}+{\text{H}}_{\text{2}}\text{O}\to \text{Cysteamine}+\text{Phosphate}$$

In order to specify the cellular compartment where the new reactions occur, we used the WoLF PSORT tool (Horton et al., 2007)^8^ and the results reported by Ghamsari et al. (2011). By providing protein sequences that are associated with the new reactions, WoLF PSORT predicted that the new reactions are localized to the cytosol.

In metabolic models, incomplete biochemical information may create gaps that form discontinuity in the network. In order to identify if any new gaps were introduced in the new model, gapFind, a COBRA command that lists root gaps, was used. The root gaps are defined as metabolites that cannot be produced in the metabolic model (Becker et al., 2007; Schellenberger et al., 2011). Using this command we found that both iRC1080 and iBD1106 models contain the same 91 root gaps. This indicates that the addition of the new metabolites and their associated reactions, did not introduce any new gaps. We note that transport reactions for the import of new metabolites into the cytosol were added.

**Figure 5:**
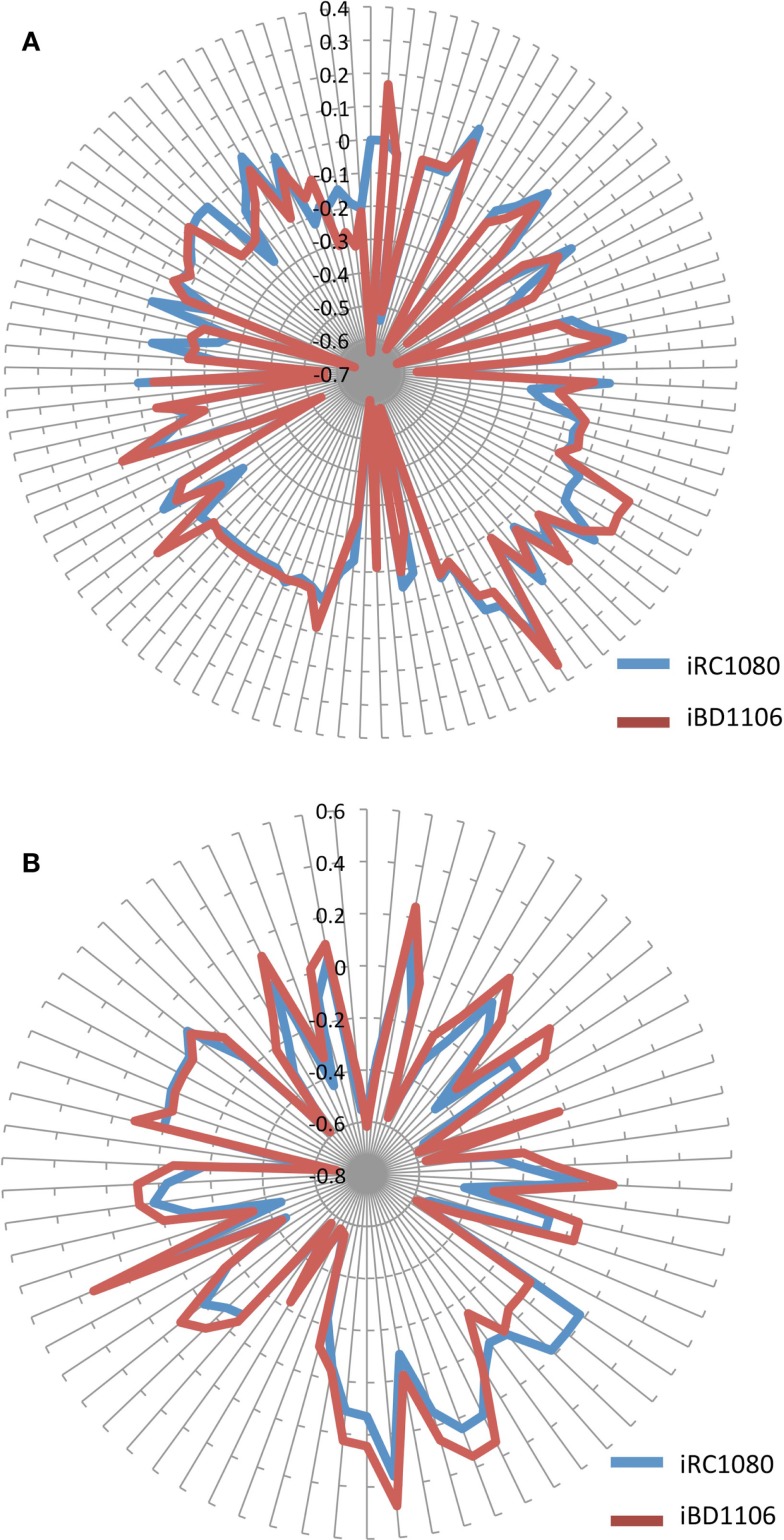
**Shadow prices of metabolites in iRC1080 and iBD1106 under different conditions for biomass maximization**. Each circle on the “radar plots” corresponds to a shadow price value, while each line extending from the center of a plot indicates a metabolite. **(A)** shows the different shadow prices and subsequently metabolic behaviors of iRC1080 and iBD1106 under light growth conditions, while **(B)** shows the different metabolic behaviors of metabolites of iRC1080 and iBD1106 under dark growth conditions.

The metabolic behavior of iBD1106 was tested under light conditions (no acetate) and dark conditions (with acetate) by carrying out flux balance analyses with the biomass as the maximized objective function. To assess the contribution that each metabolite makes to the set objective function, shadow prices for all metabolites were obtained (Tables S3 and S4 in Supplementary Material). The shadow price of a metabolite is defined as the change in an objective function with respect to flux changes of a metabolite (Varma et al., 1993; Orth et al., 2010). Shadow price allows the determination of whether a metabolite is in “excess” or is “limiting” the objective function, e.g., biomass production. Negative values are for metabolites that will decrease the objective function, positive values are for those that will increase the objective function, and values of 0 are for metabolites that will have no effect on the objective function. The comparison of shadow prices between iBD1106 and iRC1080 indicates that, for most metabolites, a large change is not observed (Figure 5; Tables S3–S5 in Supplementary Material); however, differences are observed in 105 and 70 cases under light and dark growth, respectively. Instances of such metabolites are provided in Table 4.

**Table 4:** **Examples of significant shadow prices for iRC1080 and iBD1106**.

Growth condition	Metabolite	Name	iRC1080	iBD1106
Light	4r5au	4-(1-d-Ribitylamino)-5-aminouracil	0	0.168
	5aprbu	5-Amino-6-(5′-phosphoribitylamino)uracil	−0.009	0.158
	pa1819Z18111Z	1-(9Z)-octadecenoyl,2-(11Z)-octadecenoyl-sn-glycerol3-phosphate	−0.009	−0.65
Dark	4abut	4-aminobutanoate	0.18	−0.05

## Discussion

Algae are a group of diverse photosynthetic eukaryotes, which are polyphyletic in origin (Pröschold and Leliaert, 2007). Algal lineages include the viridiplantae, which the green algae (or Chlorophyta) belong to; stramenopiles that include brown, golden, and yellow algae and diatoms; rhodophyta or the red algae; and photosynthetic alveolates that include dinoflagelates (Barton et al., 2007). Given the evolutionary distances between these lineages, significant differences in genome size and coding potential, environmental niche, and metabolic properties can be expected. Members of green algae may be aquatic or soil organisms with mixotrophic or autotrophic modes of metabolism (Pröschold and Leliaert, 2007). In addition, microalgae may or may not require co-factors for their growth. Studies on microalgal growth requirements have indicated that more than half require cobalamin (vitamine B12), while 22% require thiamine and 5% need biotin (Croft et al., 2006). Interestingly, these requirements are not reflected in algal phylogeny (Helliwell et al., 2011).

Genomic approaches powered by next-generation sequencing technologies help to improve the understanding of the encoded algal metabolic potential; however, the full characterization of algal metabolism requires phenotypic data. For instance, the metabolome of *C. reinhardtii* has been studied under a number of conditions, including sulfur deprivation (Matthew et al., 2009; Shu and Hu, 2012; Aksoy et al., 2013), nitrogen deprivation (Blaby et al., 2013; Courant et al., 2013), and response to irradiance (Mettler et al., 2014) to provide insight into regulatory and metabolic responses of the species to environmental perturbations. In addition, transcriptomics, proteomics, and metabolomics studies have guided non-targeted profiling approaches for the detection and quantification of metabolites. Those non-targeted profiling approaches have included nuclear magnetic resonance (NMR), liquid chromatography mass spectrometry (LC-MS/MS), and gas chromatography mass spectrometry (GC/MS) (Veyel et al., 2014; Wase et al., 2014). The ability to study functional responses and phenotypes has been classically limited to targeted serial studies that usually employ mutagenesis, genetic knockouts, genetic over-expression, and physiological studies (Bochner, 2003; Dent et al., 2005; Morgan et al., 2009; Tshikhudo et al., 2013; Greetham, 2014). The wealth of phenotypic information gained from the PM technology, as demonstrated in this study, can help provide more complete systems-level knowledge when combined with other omics data, and help develop and refine metabolic models.

Genome-scale metabolic networks provide predicted genotype-phenotype relationships through metabolic flux optimization-based approaches. We previously reconstructed a genome-scale model for *C. reinhardtii* (iRC1080) (Chang et al., 2011) based on literature evidence (entailing ~250 sources), structurally verified genomic evidence, and predicted gene function and cellular localization information. This model has 1,706 metabolites with 2,191 reactions. Through the pipeline that we have described in this work, we were able to expand the network significantly to include 1,959 metabolites, 2,445 reactions, and 1,106 associated genes. A clear advantage that the PM provides is functional assays for entry metabolites to inform model refinement. Whereas mass spectrometry approaches give information on intermediate- and final-level metabolites, PM assays have the unique capability, due to the accounting for entry-level metabolites, to inform more complete models from the start of metabolic pathways. PM assays and mass spectrometry can therefore be considered as complementary approaches when characterizing organisms’ metabolic profiles, with each technology refining and filling in specific gaps in metabolic models. Yet, PM’s contribution to a metabolic model’s refinement is made through a rapid, high-throughput, and convenient manner with an entire set of metabolites assayed in 5–7 days.

### New metabolites

We have identified a number of di and tripeptides, and d-amino acids that significantly expand the list of nitrogen utilization compounds in *C. reinhardtii*. While we found d-amino acids can support metabolism of *C. reinhardtii*, they may be involved in additional functions. A serine-type d-alanyl-d-alanine carboxypeptidase was found in *C. reinhardtii*’s genome that could potentially be involved in d-alanine metabolism. Serine-type d-alalyl-d-alanine carboxypeptidases have been shown to play a variety of protective roles including protection against ionic and hyperosmotic stress (Príncipe et al., 2009). A d-alanine ligase was found in *C. reinhardtii*’s genome that is potentially involved in d-alanine multimerization. Recent research using ^15^N NMR spectroscopy found that d-alanine accumulated in plants during UV exposure and this finding is supported by previous research under various stress signals (Monselise et al., 2014). Therefore, the possibility that d-amino acids might have additional cellular functions in *C. reinhardtii*, aside from providing a source of nitrogen, can be a subject of future investigations.

*Chlamydomonas reinhardtii* is known to be able to use a variety of amino acids as a sole nitrogen source as long as acetate is present (Munoz-Blanco et al., 1990). In *C. reinhardtii*, arginine is the only amino acid known to be imported with high affinity; the rest are believed to be deaminated extracellularly (Kirk and Kirk, 1978) or transported passively (Zuo et al., 2012). We note that a search in the literature for d-amino acid transports has not provided any information on the mode of transport for this class of amino acids in *C. reinhardtii*, nor is it known if the *C. reinhardtii* deaminase can deaminate d-amino acids. However, *C. reinhardtii* has been shown to exhibit amino acid racemerase activity (Takahashi et al., 2012), which could explain the ability to assimilate d-amino acids intracellularly. This also provides indirect evidence that these amino acids may be absorbed or transported into the cell for conversion to their L counterparts. A biological function for d-amino acids has not been clearly defined; however, d-alanine and d-aspartate were detected in algae using a reversed-phase HPLC; d-alanine was present in some marine diatoms while d-aspartate was found in all the selected freshwater green microalgae and marine diatoms (Yokoyama et al., 2003).

In many microbes, dipeptides are imported into the intracellular compartment before they are eventually hydrolyzed. For instance, *Francisella tularensis* relies on an amino acid transporter of the major facilitator superfamily of secondary transporters for transporting amino acids intracellularly. Furthermore, dipeptides containing asparagine were effective at restoring cellular multiplication in the infection cycle of a *F. tularensis* mutant that lacked that essential amino acid transporter (Gesbert et al., 2014). In this study, a variety of dipeptides were found to promote heterotrophic respiration in *C. reinhardtii*. The latest version of *C. reinhardtii*’s genome contains a gene annotated as a peptide hydrolase Cre02.g078650.t1.3. We note that the detected utilization of the dipeptides is not without sequence specificity as 159 out of 267 of the dipeptides and 9 out of 14 of the tripeptides did not result in positive assay results.

From these newly identified metabolites, three phosphorus compounds were found: (1) cysteamine-*S*-phosphate (C_2_H_7_NO_3_PS), which is an organic phosphorothioate anion that is derived from deprotonation of thiophosphate OH groups and protonation of the amino group, (2) thiophosphate (or phosphorothioate), and (3) dithiophosphate, which is the product of the reaction of a base with phosphorus pentasulfide.

The only new sulfur source that was identified, tetrathionate, is a sulfur oxoanion and is derived from the compound tetrathionic acid and is commonly found in soils. It is a key intermediate in the oxidation of various reduced inorganic sulfur compounds. Several species of bacteria including *Salmonella enterica* (Winter et al., 2010) and *Acidithiobacillus ferrooxidans* (Rohwerder et al., 2003; Holmes and Bonnefoy, 2007; Chen et al., 2012) are known to be able to assimilate tetrathionate. Strains of *A. ferrooxidans* overexpressing tetrathionate hydrolase (tetH) were found to grow better on both sulfur and tetrathionate. In the archeon *Acidianus hospitalis*, tetrathionate is secreted to form filaments from tetrathionate homomultimers (Krupovic et al., 2012). These remarkable filaments are believed to play a role in sulfur metabolism and adaptation to *A. hospitalis*’s extreme environment. Prokaryotes have also been shown to use tetrathionate as an electron acceptor in cobalamin (coenzyme B12) synthesis (Roth et al., 1996). Sulfur is commonly assimilated as reduced sulfur for most living organisms, but bacteria are known to reduce tetrathionate, thiosulfate, sulfite, sulfur, and dimethyl sulfoxide in dissimilatory reactions as well (Barrett and Clark, 1987). Tetrathionate is often used as an electron sink for oxidative phosphorylation (Chen et al., 2012). Bacteria that are known to respire using tetrathionate are often found to have the capability of reducing thiosulfate as well, but thiosulfate is not found to be reduced among organisms that do not respire thiosulfate (Rohwerder et al., 2003). Considering that *C. reinhardtii* is a soil organism, the ability to assimilate this compound is likely to provide an important survival advantage in *Chlamydomonas*’ natural life cycle.

### iBD1106 model vs. iRC1080

Different behaviors can be observed for iBD1106 than those for iRC1080 under different conditions. When the biomass production was set as the objective function, a differential change can be noticed as a result of growth conditions. The addition of the new nitrogen sources (d-amino acids, dipeptides, and tripeptides) has a significant and differential effect on the shadow prices of metabolites under light and dark conditions for biomass production (Figures 5A,B, respectively).

Under light growth, the d-aspartate in iBD1106 showed significant effect on the behavior of the chloroplastic metabolites of the riboflavin pathway. In iBD1106, d-aspartate is converted into l-aspartate through racemase, and l-aspartate can be produced through hydrolysis of its dipeptides (Asp–Leu, Asp–phe, Pro–Asp, Asp–Ala, Asp–Gln, and Asp–Gly). Also the oxidation of d-asparagine produces d-aspartate as oxo-carboxylate (Eq. 1). The addition of l-aspartate increases its consumption in purine metabolism, which yields to more production of 2,5-Diamino-6-hydroxy-4-(5′-phosphoribos ylamino)-pyrimidine (25dhpp). The latter can be converted into 5-Amino-6-(5′-phosphoribosylamino)uracil (5apru) in the riboflavin metabolism resulting in an excess of 4-(1-d-Ribitylamino)-5-aminouracil (4r5au) and 5aprbu, with shadow prices of 0.168 and 0.158, respectively. Those results were not observed in iRC1080.

Another example of model discrepancy under light growth is the effect of adding d-serine reactions in iBD1106. Addition of d-serine limited the availability of the metabolite 1-(9Z)-octadecenoyl,2-(11Z)-octadecenoyl-sn-glycerol-3-phosphate (pa1819Z18111Z) (shadow price −0.009 in iRC1080 and −0.65 in iBD1106). This metabolite is produced and consumed by the reactions of glycerolipid metabolism for the production of Palmitoyl-CoA (n-C16:0CoA) (pmtcoa). The addition of l-serine in iBD1106 results in more consumption of pmtcoa in the sphingolipid metabolism through the reaction serine C-palmitoyltransferase (SERPT) that produces 3-dehydrosphinganine.

Under dark growth conditions, 4-aminobutanoate was in excess in iRC1080 and became limiting in iBD1106 with shadow price values of 0.18 and −0.05, respectively. The reason for this limiting availability is the addition of d-histidine and d-glutamate dipeptides hydrolysis reactions, e.g., Ala–His, and inversion into l-histidine and l-glutamate through a racemase. This addition increases the consumption of l-glutamate and l-histidine along with 4-aminobutanoate in glutamate and arginine and proline metabolisms, respectively. Moreover, the dark growth condition did not affect the behavior of 4-aminobutanoate significantly in iBD1106; however, in iRC1080 it was shifted from a limiting metabolite (−0.07) into an excess metabolite (0.18) (Table 4). The excessiveness of 4-aminobutanoate in iRC1080 under dark conditions might be related to the high consumption of NADPH under dark growth conditions. In proline metabolism, NADPH and 4-aminobutanoate are consumed more rapidly in dark than that in light conditions. As such, the addition of d-histidine and d-glutamate compensates the effect of growth under dark in the proline metabolism.

## Conclusion

Phenotypic profiling has tremendous utility in modeling and understanding algal metabolism and is essential in elucidating genotypic differences in algae and the effects of environmental conditions on metabolism. The method presented here demonstrates the first reproducible study utilizing PM assays in profiling microalgae using *C. reinhardtii* as a model. We observed positive growth on 148 nutrients (one positive assay for C-source utilization, four positive assays for the S-source and P-source utilization, and 139 positive assays for N-source utilization). The wealth of phenotypic data can be used along with other references to compare organisms with known mutants or unknown isolates. This wealth of information will also shed light on new and novel metabolic pathways. The substrate utilization information and the newly identified metabolites were used for metabolic network expansion and refinement of the iRC1080 metabolic model. The study also provides a framework to bridge the missing links between genomics and metabolomics in microalgae. The described work provides an excellent method for the initial characterization of newly isolated or uncharacterized strains of algae. This combination of high-throughput phenotypic screening with metabolic modeling can allow for rapid refinement of existing metabolic network models as demonstrated and also provides biochemical evidence to support *de novo* reconstruction of new algal models.

## Conflict of Interest Statement

The authors declare that the research was conducted in the absence of any commercial or financial relationships that could be construed as a potential conflict of interest.

## Supplementary Material

The Supplementary Material for this article can be found online at http://www.frontiersin.org/Journal/10.3389/fbioe.2014.00068/abstract

**Data Sheet S1:**

Table S1 **PM assays for PM01 to PM04 and PM06 to PM08, positive assays are shaded with yellow**.

Table S2 **A table of new metabolites that were added to generate iBD1106 along with their genetic annotations**.

Table S3 **Shadow prices for metabolites of iRC1080 and iBD1106 when biomass was set as objective function under growth with light and no acetate**.

Table S4 **Shadow prices for metabolites of iRC1080 and iBD1106 when biomass was set as objective function with growth under dark with acetate**.

Table S5 **Shadow prices for new metabolites in iBD1106 when Biomass was set as the objective function (growth under light without acetate and under dark with acetate)**.

Data Sheet S2 ***Chlamydomonas reinhardtii* metabolic network model, iBD1106, in SBML format**.

Figure S1 **Phenotype microarray results for plates 1–4, and 6–8**.

## Footnotes

^1^http://www.genome.jp/kegg/

^2^http://metacyc.org/

^3^http://www.phytozome.net

^4^http://pfam.xfam.org/search

^5^http://www.ebi.ac.uk/interpro/

^6^http://www.uniprot.org/

^7^https://blast.ncbi.nlm.nih.gov/Blast.cgi

^8^http://www.genscript.com/psort/wolf_psort.html
