# Supplementary material for: Microalgal Metabolic Network Model Refinement through High-Throughput Functional Metabolic Profiling
Source: Front Bioeng Biotechnol. 2014 Dec 10;2:68. doi: 10.3389/fbioe.2014.00068 (PMC4261833; doi:10.3389/fbioe.2014.00068)
Supplement: Figure S1 — Phenotype microarray results for plates 1–4, and 6–8. [file Image_1.PDF]

A

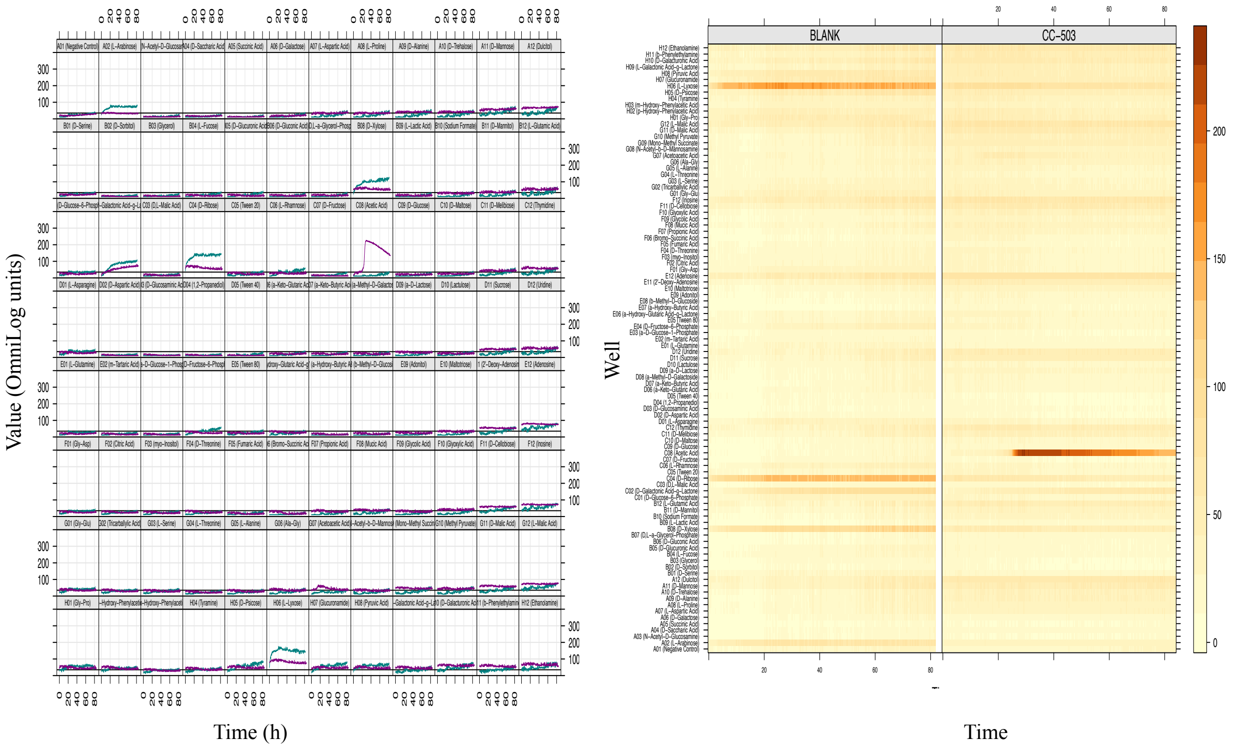
Value (OmniLog units)
Time (h)
BLANK
CC-503
Well
Time

B

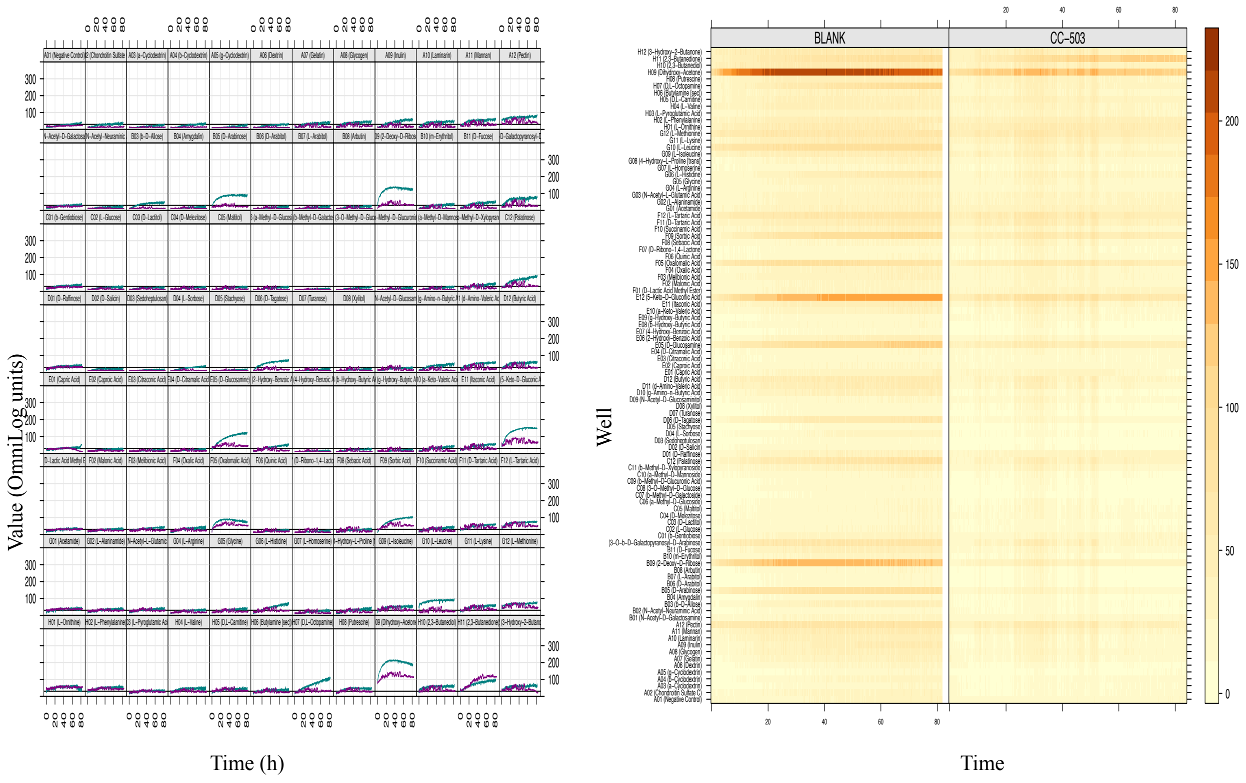
Value (OmniLog units)
Time (h)
BLANK
CC-503
Well
Time

C

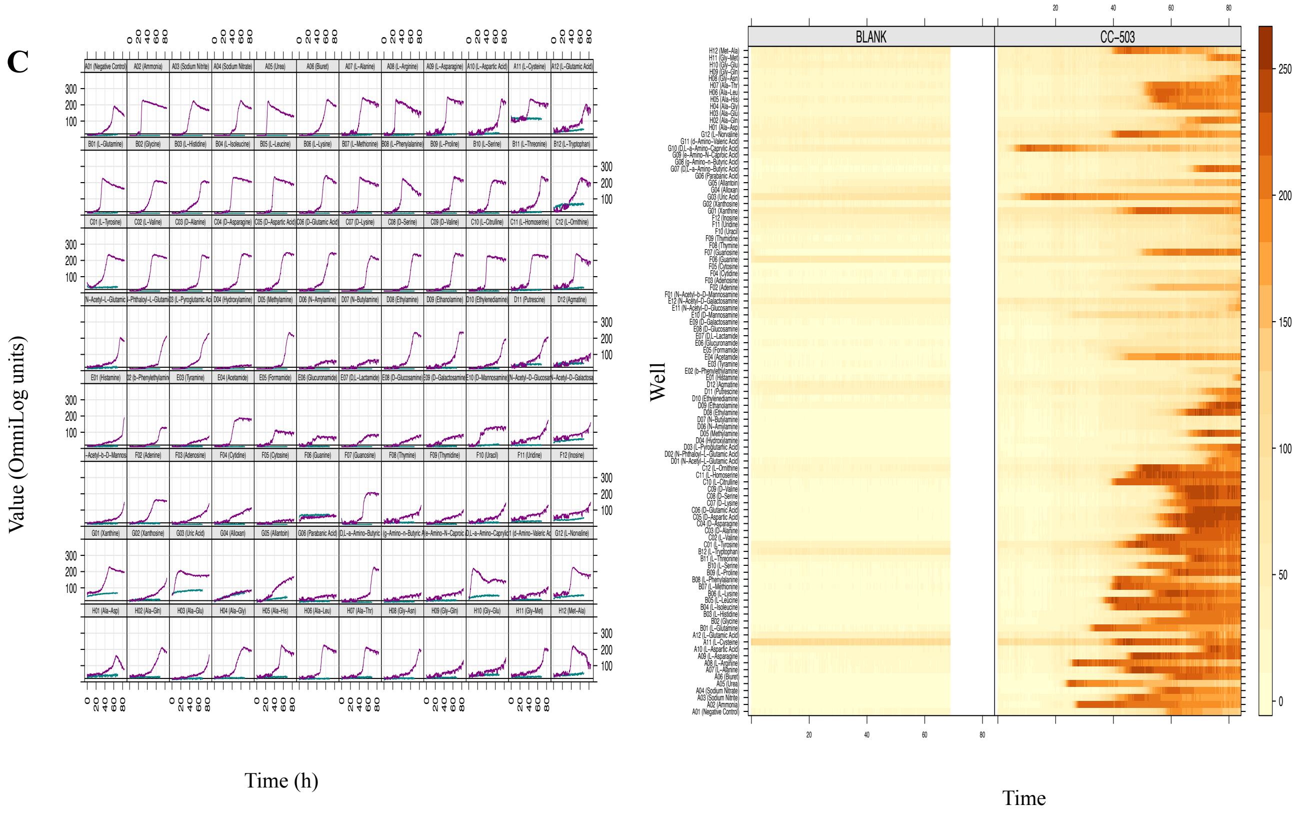
Value (OmniLog units)
Time (h)
BLANK
CC-503
Well
Time

D

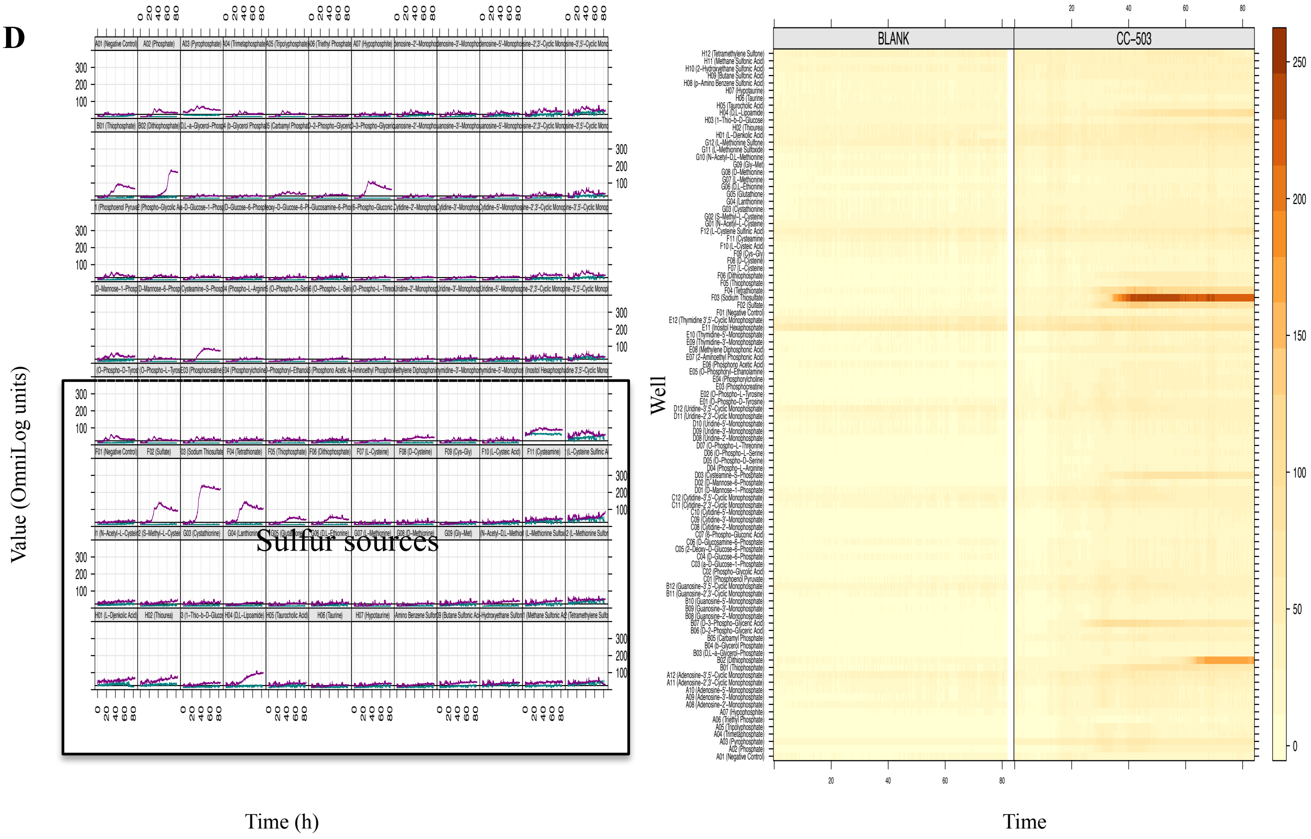
Value (OmniLog units)
Sulfur sources
Time (h)
BLANK
CC-503
Well
Time

E

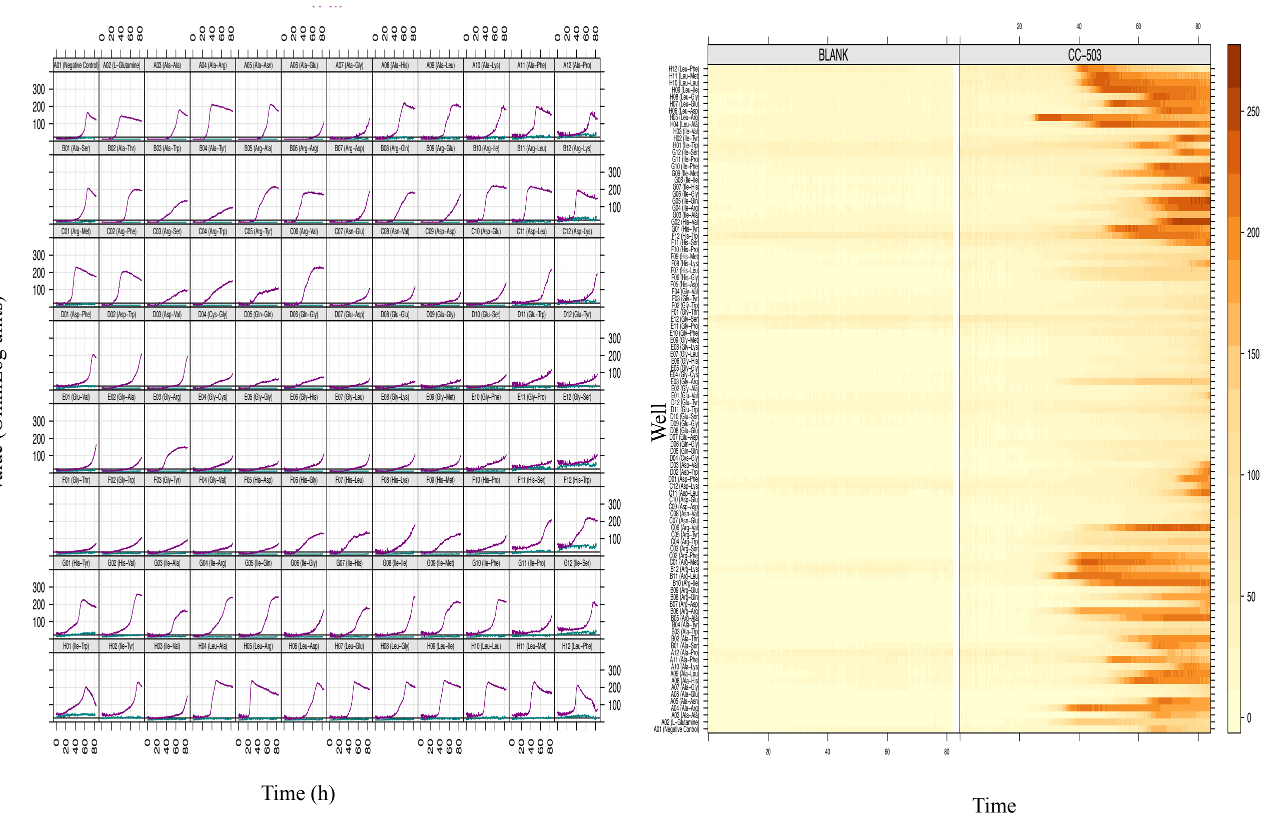

Value (OmniLog units)
Time (h)
BLANK
CC-503
Well
Time

F

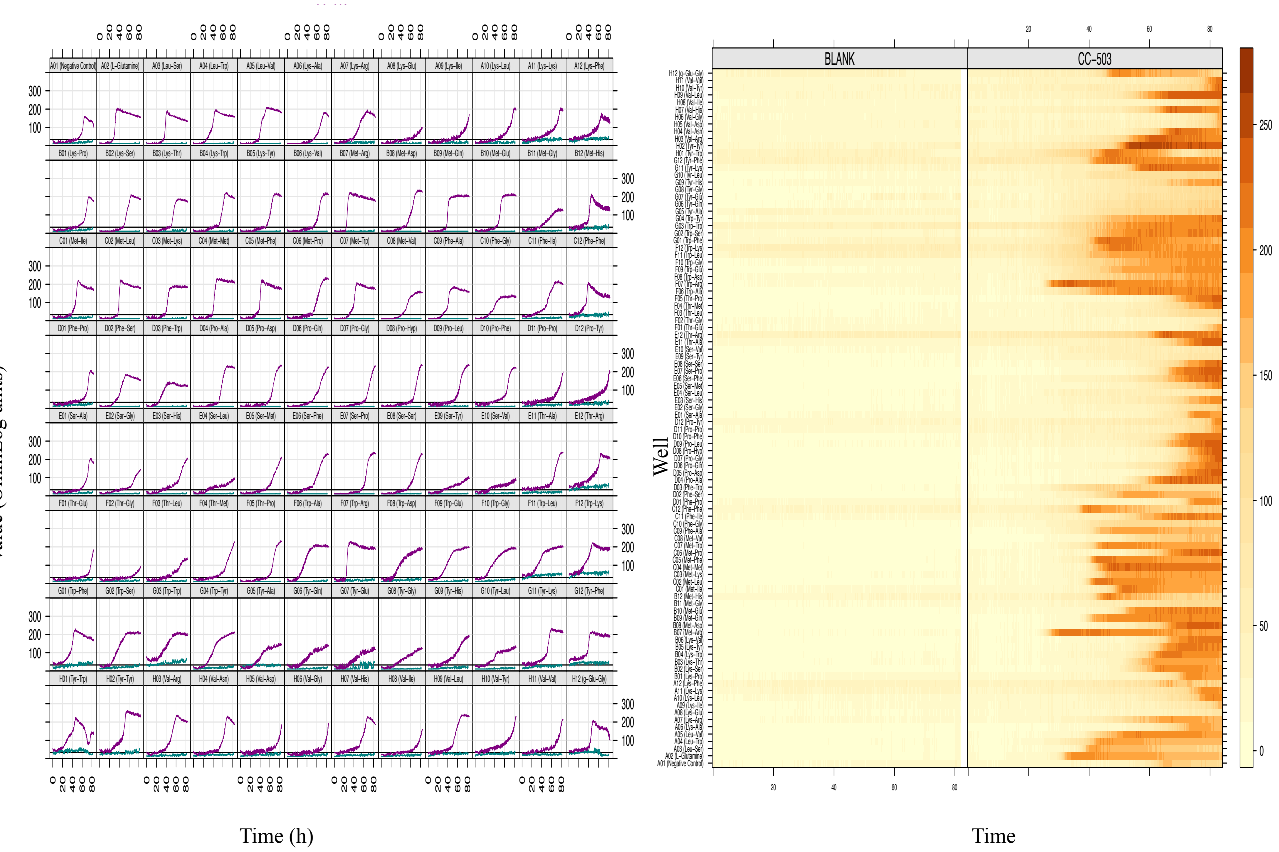

Value (OmniLog units)
Time (h)
BLANK
CC-503
Well
Time

G

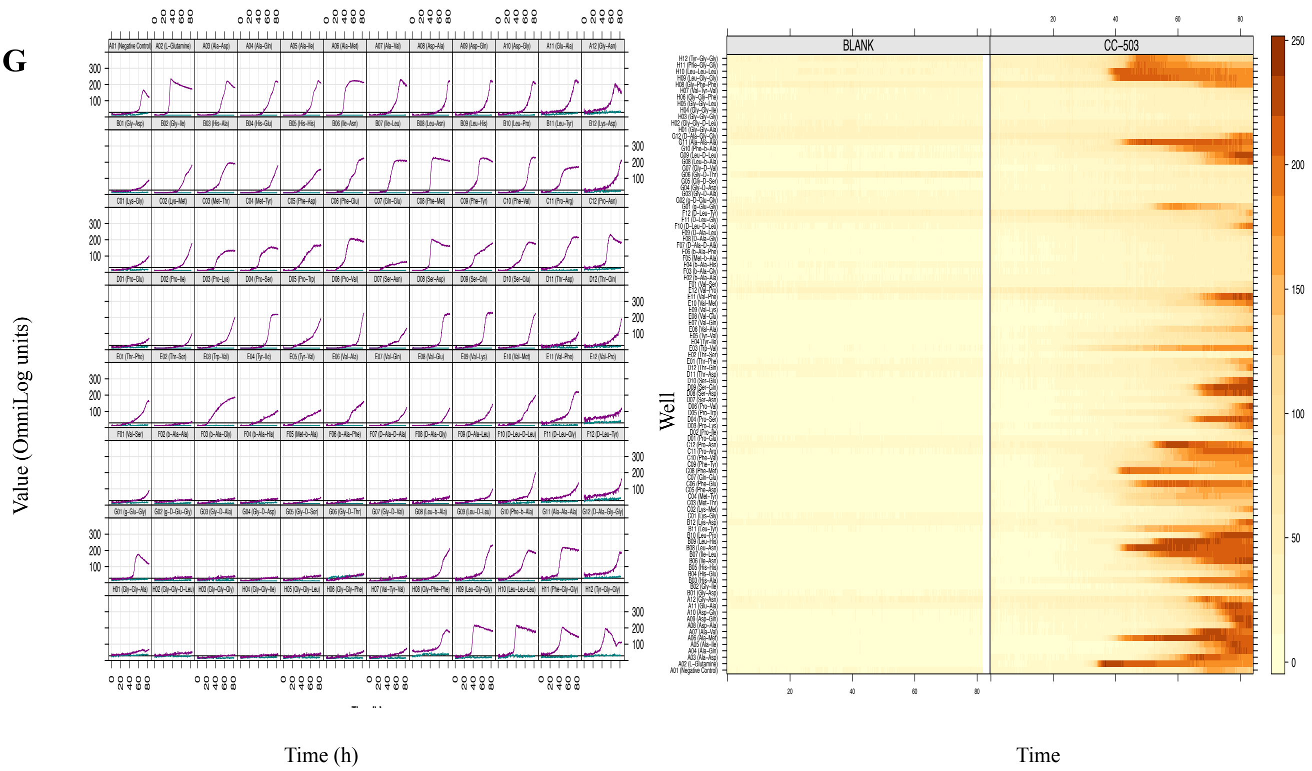

Supplementary Figure 1: Phenotype microarray profiling of *C. reinhardtii* metabolism. Each panel represents respiration levels over time. XY-plot and level plots of each plate are shown. (A) to (G) show assays observed in the seven plates (PM01 to PM04, and PM06 to PM08). (A) and (B) for carbon sources, (C) nitrogen sources), (D) phosphorus and Sulfur sources, and (E) to (G) nitrogen sources from peptides. Each panel in the figure is a 12x8 array of 96-well plate representations. Curves within each well represent dye conversion by reduction (y-axis) in time (x-axis). PM curves from CC-503 and blanks are indicated by color (teal color represents blank and purple color represents CC-503). For the level-plots, each respiration curve is displayed as a thin horizontal line (shading change from light yellow to dark orange or brownish based on the level of respiration measurement values. Metabolites utilized by *C. reinhardtii* (CC-503) and blank plate basal reactions are shown with positive assay being distinguished by their magnitude and kinetics exceeding both the negative control and blank plates.
